# Prevalence and Temporal Trends of Deformational Plagiocephaly and Brachycephaly in Finland: A Population-Based Register Study

**DOI:** 10.1097/SCS.0000000000012892

**Published:** 2026-05-22

**Authors:** Pia Vuola, Arja Heliövaara, Turkka Kirjavainen, Niklas Pakkasjärvi, Annukka Ritvanen, Erkki Tukiainen, Mika Gissler

**Affiliations:** * Department of Plastic Surgery, Helsinki University Hospital and University of Helsinki, The Cleft Palate and Craniofacial Center, Helsinki, Finland; † Department of Pediatrics, Helsinki University Hospital and University of Helsinki, Helsinki, Finland; ‡ Department of Pediatric Surgery, Helsinki University Hospital and University of Helsinki, Finland; § THL Finnish Institute for Health and Welfare, Register of Congenital Malformations, Helsinki, Finland; ∥ Department of Plastic Surgery, Helsinki University Hospital and University of Helsinki, Helsinki, Finland; ¶ Department of Data and Analytics, THL Finnish Institute for Health and Welfare, Helsinki, Finland; # Region Stockholm, Academic Primary Health Care Centre, Stockholm, Sweden; ** Department of Molecular Medicine and Surgery, Karolinska Institutet, Stockholm, Sweden

**Keywords:** Craniosynostosis, deformational brachycephaly, deformational plagiocephaly, prevalence

## Abstract

This nationwide population-based study evaluated national trends in the prevalence of deformational plagiocephaly (DP) and deformational brachycephaly (DB) in Finland, using comprehensive registry data. We analyzed 1,453,780 births in Finland between 1987 and 2010. We identified 467 children diagnosed with DP or DB, including one stillbirth. Data were obtained from national health registers and supplemented with individual medical record reviews. Diagnoses were confirmed or corrected, and patients with craniosynostosis were excluded. Although Finland did not launch a formal “Back to Sleep” campaign against sudden infant death syndrome, supine sleep recommendations were gradually adopted and formally revised in 2000. Between 1987 and 2010, DP and DB prevalence rose from 1.7 per 100,000 live births in 1987 to 136 per 100,000 live births in 2010 (*P*<0.001), with the most pronounced increase occurring after 2000. In the group of children with moderate-to-severe deformities, 62% had no additional anomalies, 29% had 1 to 3 anomalies, and 9% were syndromic. In this group, male predominance (69%) and right-sided occipital flattening (56%) were consistent with findings reported in previous studies. Additionally, the proportion of twins was higher (6.7%) than the national twin birth rate during the same period (2.7%). This study demonstrated a strong temporal correlation between the rising prevalence of DP and DB and evolving supine sleep recommendations in Finland. By the end of the study period, DP and DB prevalence had surpassed that of craniosynostosis, mirroring international trends, although this transition occurred later in Finland than in many other countries.

Deformational plagiocephaly (DP) and deformational brachycephaly (DB) are cranial shape abnormalities resulting from external forces acting on an infant’s skull during pre- or postnatal growth.^1^ Although deformities result from mechanical pressure, their development is influenced by both intrinsic (male sex, prematurity) and extrinsic (prolonged supine positioning, multiple births) risk factors.^2,3^ Deformational plagiocephaly typically involves unilateral occipital flattening and may be associated with external ear position asymmetry, facial asymmetry, and cranial compensatory changes in more severe cases. Deformational brachycephaly presents as symmetrical occipital flattening and increased cephalic width, and often co-occurs with posterior DP.^4,5^ Anterior (frontal) DP, although less common, results from frontal compression.^6^

These conditions differ from craniosynostosis, which involves prematurely fused cranial sutures, resulting in a characteristic cranial shape depending on which suture is affected.^7^ The Argenta clinical classification can be used to evaluate the diagnosis and severity of DP and DB.^8^ The classification is based on visual assessment rather than imaging or anthropometric measurements and is useful in clinical evaluation and follow-up.^9,10^

The incidence of localized cranial flattening in newborn singletons, assessed using anthropometric measurements in maternity wards, was reported to be 13% in the United States (n=201; Boston), whereas a Finnish study found a prevalence of 7.7% (n=155; Oulu).^11,12^ Localized cranial flattening at birth, cervical imbalance, and head position preference may be risk factors for DP; however, they are not necessarily indicative of DP if motor development progresses normally.^13^

Notably, DP typically becomes more common between 1 and 4 months of age. In a cohort of healthy, normally developing children (n=200), the prevalence of DP and/or DB was 16.0% at 6 weeks, increased to a peak of 19.7% at 4 months, and subsequently decreased to 9.2% at 8 months, 6.8% at 12 months, and 3.3% at 24 months of age.^14^ Global prevalence estimates of DP or DB range from 10% to 15% to as high as 48% in infants aged <12 months, with the highest rates observed in populations with high adherence to supine sleep positioning.^10,15,16^

SIDS refers to the sudden, unexpected death of an infant aged <1 year that remains unexplained after a thorough case investigation.^17^ The prone sleeping position is a significant risk factor for SIDS.^18^ Following the introduction of large “Back to Sleep” campaigns in many countries in the late 1980s and early 1990s, together with the 1992 American Academy of Pediatrics recommendation for side or supine infant sleep to reduce the risk of SIDS, a significant increase in DP and DB prevalence was observed across several countries.^19,20^ Although the association between supine sleeping and cranial deformity remains observational, it has been consistently reported.^21,22^

In Finland, SIDS rates were lower in the 1980s than in other Nordic countries, and no formal nationwide campaign promoting supine sleep was implemented in the early 1990s.^23^ Sleep position recommendations evolved more gradually in Finland, with side sleeping commonly advised in maternity clinics until the early 2000s, after which supine sleeping was increasingly adopted following a guideline revision.^24^

Despite the increasing awareness of cranial shape deformities, population-based data on their long-term trends remain scarce. To address this gap, we conducted a nationwide registry-based study in Finland between 1987 and 2010 to examine temporal trends in DP and DB prevalence. We also assessed their relationship with evolving sleep-positioning recommendations and compared these trends with those observed for craniosynostosis during the same period.

## METHODS

### Study Setting and Data Sources

Finland is a high-income country, with a population of approximately 5.6 million. Maternal and child health care is publicly funded, free or heavily subsidized, universally accessible, and almost exclusively utilized. The evaluation and treatment of craniosynostosis are centralized at 2 university hospitals (Helsinki and Oulu), whereas cranial deformities are assessed in pediatric outpatient clinics at secondary hospitals and at all 5 university hospitals nationwide.

All births, congenital malformations, and specialized health care encounters are recorded in nationwide registers maintained by the Finnish Institute for Health and Welfare. Statistics Finland records the mortality data. Unique personal identity codes enable reliable linkages across registries, hospital records, and health outcomes. These data are available for scientific research through approved applications and provide virtually complete national coverage.^25^

### Study Population and Case Ascertainment

This study forms part of a nationwide investigation of craniosynostosis and cranial deformities in Finland between January 1, 1987, and December 31, 2010. All live births, stillbirths, and terminations of pregnancy for fetal anomaly (TOPFA) during the study period were screened using national health registers. During this period, 1,447,958 live births, 5822 stillbirths, and 5,363 TOPFAs were reported. The mean annual number of live births was 60,331 (range, 55,333–66,642).

The patients were identified using predefined ICD-9 and ICD-10 diagnostic and procedure codes, as shown in Supplementary Table 1 (Supplemental Digital Content 1, http://links.lww.com/SCS/J546: Table 1A, Diagnostic codes used in the study; Table 1B, Procedure codes used in the study).

Data were obtained from the Register of Congenital Malformations, Medical Birth Register, Care Register for Health Care (formerly the Hospital Discharge Register), Register of Small Preterm Infants, and Register of Induced Abortions. Cause-of-death data were retrieved from Statistics, Finland. Registry data were collected in 2013 to ensure complete ascertainment of cases. In addition, the diagnoses recorded in the national registers were supplemented and cross-validated using independently collected patient records from the Helsinki University Hospital and Helsinki City Hospital Aurora.

### Diagnostic Validation

All data were linked using personal identity codes for the mothers and live-born children. A total of 1878 medical records were reviewed to confirm or correct the diagnoses of craniosynostosis or cranial deformities. Diagnostic confirmation was based on patient records, including clinical examinations, radiological findings, surgical reports, and photographs. Records were independently reviewed by a craniofacial surgeon (P.V.) and clinical geneticist (A.R.), with discrepancies resolved by consensus. The Register of Congenital Malformations is legally authorized to retrieve medical records from all hospitals in Finland for diagnostic validation. All confirmed cases were included regardless of age at diagnosis, and the prevalence was calculated using the birth cohort.

The craniosynostosis cohort has been previously described.^26^ A total of 877 craniosynostosis cases were confirmed: 864 live births, 5 stillbirths, and 8 TOPFAs. The present study included 467 children with DP or DB, comprising 466 live births and one stillbirth (Fig. 1).

**FIGURE 1:**
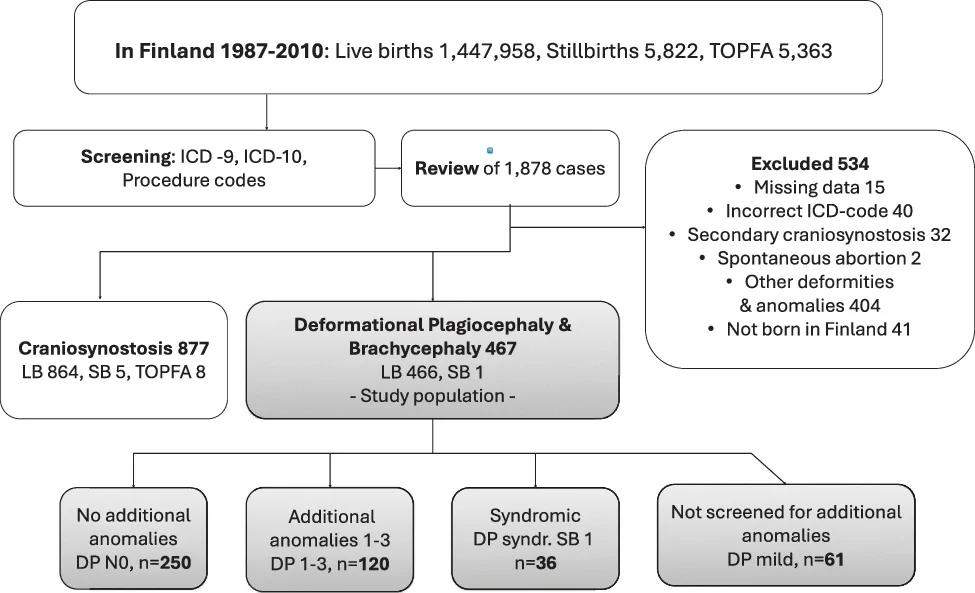
Study cohort flow chart.

### Classification of DP and DB

Confirmed cases included anterior and posterior DP and DB, with no evidence of craniosynostosis. Children were identified through referrals from primary care to secondary or tertiary outpatient clinics for the evaluation of abnormal head shape or during evaluations for unrelated anomalies. Initially, TOPFA cases were included; however, as cranial deformities identified on postmortem examination were secondary to severe primary anomalies, terminated pregnancies were excluded, while stillbirths were retained.

Severity was assessed using the Argenta clinical classification system.^8^ Mild posterior DP cases (Argenta type I), defined as localized unilateral occipital flattening without compensatory cranial changes, and mild DB cases restricted to the central posterior skull (Argenta type I) were difficult to differentiate from normal variations in skull shape based solely on retrospective medical records. Consequently, these cases were excluded from the selected prevalence analyses, and the associated anomalies were not evaluated in this group.

Moderate-to-severe posterior DP Argenta types II to V, characterized by progressive asymmetry of ear position, frontal protrusion, facial asymmetry, and compensatory cranial changes, could not be reliably distinguished retrospectively and were therefore analyzed as a combined group. Moderate-to-severe DB cases (Argenta types II–III), characterized by widening of the posterior skull lateral to the central depression (type II) with additional compensatory temporal or vertical growth in type III, as well as anterior DP cases, were also included in this combined group.

Additional anomalies were identified in this group by using data from the Register of Congenital Malformations. Data on additional anomalies were reviewed and corrected in the same manner as for the diagnoses. Patients were classified as syndromic if a named syndrome (excluding craniosynostosis syndromes) was recorded, or if 4 or more anomalies were present.

### Statistical Analysis

The prevalence rates of DP and DB were calculated per 10,000 and 100,000 live births with 95% confidence intervals using binomial proportion methods. Temporal trends were assessed using the χ^2^ test. Linear regression models using ordinary least squares were fitted separately for DP and DB (including mild cases) and for craniosynostosis, with annual prevalence as the dependent variable and calendar year as a continuous predictor. Locally estimated scatterplot smoothing (Loess) curves were used to visualize nonlinear trends. Statistical significance was set at *P*<0.05. Analyses were performed using R, version 4.5.1.

### Ethics Approval

The study was approved by the Finnish Institute for Health and Welfare. Additional permissions were obtained from the Helsinki University Hospital, Helsinki City Hospital, and Statistics Finland. This study was conducted in accordance with the principles of the Declaration of Helsinki.

## RESULTS

In total, 467 children born in Finland between 1987 and 2010 were diagnosed with DP or DB and were recorded in nationwide registers. Of these, 406 were classified as moderate-to-severe posterior DP types II to V, moderate-to-severe DB types II to III, or anterior DP (combined group); 405 were live births and one was a stillbirth (with a named syndrome other than craniosynostosis), and all were screened for additional anomalies. An additional 61 live-born children with mild posterior DP type I or mild DB type I were identified but were not screened for additional anomalies. All patients independently identified in the hospital records of Helsinki University Hospital and Helsinki City Hospital were documented in the Care Register for Health Care.

### Patient Characteristics of the Combined Group)

Of the 405 live-born patients in the combined group (moderate-to-severe posterior DP types II-V, moderate-to-severe DB types II-III, and anterior DP), 68% were male. Posterior DP was the most common diagnosis (90%; n=364), followed by symmetrical DB (7%; n=28), and anterior (frontal) DP (3%; n=13). Among cases of posterior DP, occipital flattening occurred on the right side in 56% and on the left side in 38%, and laterality was unclassified in 6% of cases. Of the patients, 61.7% (n=250) had no additional anomalies, 29.6% (n=120) had 1 to 3 anomalies, and 8.7% (n=35) were syndromic, defined as having a named syndrome other than craniosynostosis or 4 or more additional anomalies (Fig. 2). The characteristics of patients in the combined group are shown in Supplementary Table 2 (Supplemental Digital Content 2, http://links.lww.com/SCS/J547).

**FIGURE 2:**
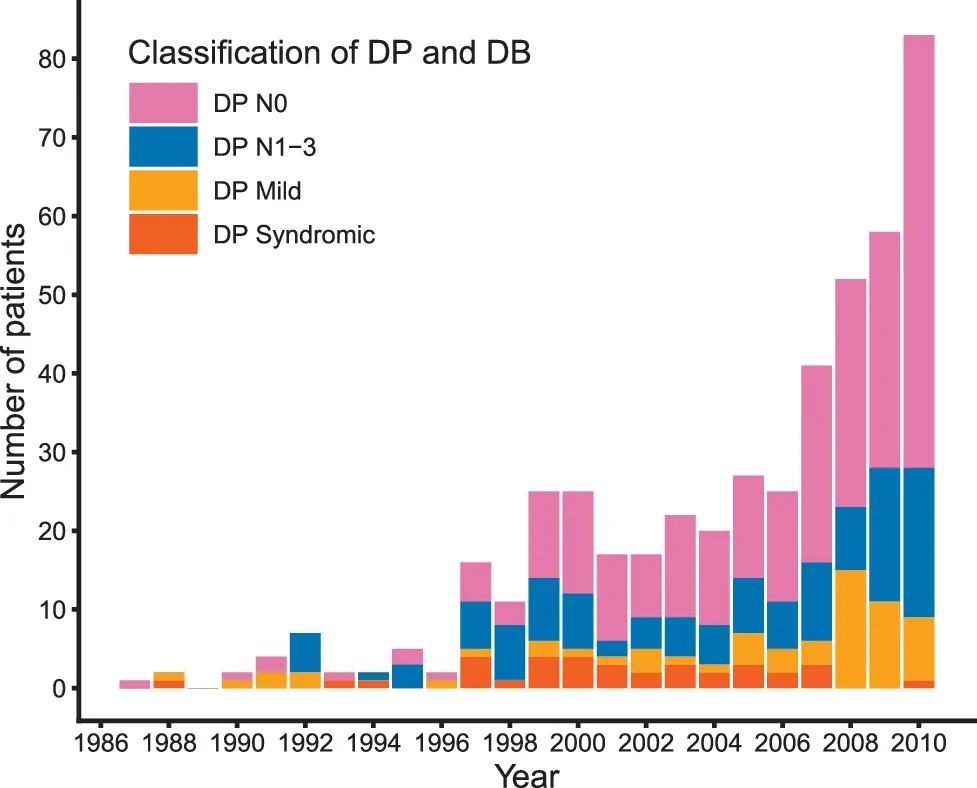
Absolute numbers of live-born patients (n=466) diagnosed with deformational plagiocephaly (DP) or deformational brachycephaly (DB) in Finland. Patients were categorized as mild posterior DP or mild DB (DP Mild), and as moderate-to-severe posterior or anterior DP or DB, stratified into those without additional anomalies (DP N0), with 1 to 3 additional anomalies (DP N1–3), or syndromic patients (DP Syndromic). Mild cases were not screened for additional anomalies.

#### Patients Without Additional Anomalies

Of 405 live births, 250 (61.7%) had no additional anomalies. In this subgroup, 69% of the patients were male. Posterior DP was present in 88% (n=220) of patients, with right-sided flattening in 55.5%, left-sided flattening in 38.6%, and unspecified laterality in 5.9%. Deformational brachycephaly was present in 7% (n=18) and anterior DP in 5% (n=12) of patients.

#### Patients With 1–3 Additional Anomalies

A total of 120 patients (29.6%) had between 1 and 3 additional anomalies. Of these, 70% were male. Posterior DP was identified in 92.5% (n=111) of patients, with right-sided flattening in 59.5%, left-sided flattening in 36.0%, and unspecified laterality in 4.5%. Only one patient (0.8%) had anterior DP and 6.7% (n=8) had DB.

#### Syndromic Patients

Thirty-five patients (8.7%) among the live births were classified as syndromic. These cases were defined by the presence of a named syndrome (other than craniosynostosis) or 4 or more additional anomalies. Of these, 57% were male. Posterior DP was observed in 94% (n=33) of patients, with right-sided flattening in 46%, left-sided flattening in 36%, and unspecified laterality in 18%. None of the patients had anterior DP, whereas DB was present in 6% of the patients (n=2).

Among syndromic patients, skull shape abnormalities were commonly associated with underlying conditions, such as muscle hypotonia or treatment-related positional effects due to other anomalies (eg, cardiac or orthopedic conditions).

#### Stillbirths

Cranial deformities and skull shape abnormalities were common among stillborn infants recorded in the Register of Congenital Malformations. Craniosynostosis cases were identifiable in the postmortem reports (n=5), whereas other cranial shape abnormalities were generally associated with underlying diseases or additional anomalies. However, one infant presented with a clearly recognizable posterior DP classified as Argenta type V without synostosis and had a diagnosed syndrome unrelated to craniosynostosis.

#### Twins

Twins accounted for 6.7% (n=27) of the live birth cohort. The highest proportion of twins was observed in patients without additional anomalies (8%; n=20). These proportions were higher than the expected national rate (2.7%), suggesting a possible association between twin gestation and DP or DB development.

### DP and DB Prevalence

The prevalence of the combined group (moderate-to-severe posterior DP types II-V, moderate-to-severe DB types II-III, and anterior DP) increased from 1.7 per 100,000 live births in 1987 (95% CI, 0.04–9.3; approximately 1 per 60,000) to 123 per 100,000 in 2010 (95% CI, 96–154; approximately 1 per 800) (*P*<0.001, χ^2^ test for trend). The increase was most pronounced among children without additional anomalies, but remained significant across all groups, including the syndromic group (*P*=0.016) (Fig. 3).

**FIGURE 3:**
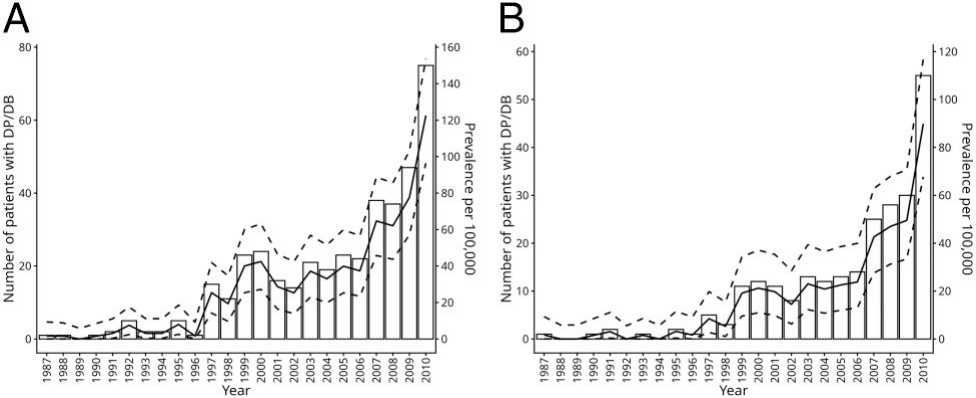
Absolute annual numbers and live birth prevalence of DP and DB excluding mild cases. The black line represents annual prevalence, with 95% confidence intervals (CIs) shown as dotted lines. The right y-axis indicates prevalence per 100,000 live births, whereas the columns and left y-axis represent absolute case numbers per year. (A) Combined total of patients without additional anomalies, with 1 to 3 additional anomalies, and syndromic patients (n=405). (B) Patients without additional anomalies (n=250). DP, deformational plagiocephaly; DB, deformational brachycephaly.

### DP and DB Prevalence Compared With Craniosynostosis

Including mild cases (DP type I and DB type I; n=61) in the combined group, the total prevalence of DP and DB in 2010 was 136 per 100,000 live births (95% CI, 108–168; approximately 1 per 735).

The prevalence of craniosynostosis increased from 50 per 100,000 live births in 1987 (95% CI, 34–72; approximately 1 per 2000) to 75 per 100,000 live births in 2010 (95% CI, 55–100; approximately 1 per 1333).^26^ In 2008, the absolute number and prevalence of DP and DB surpassed those of craniosynostosis (Fig. 4).

**FIGURE 4:**
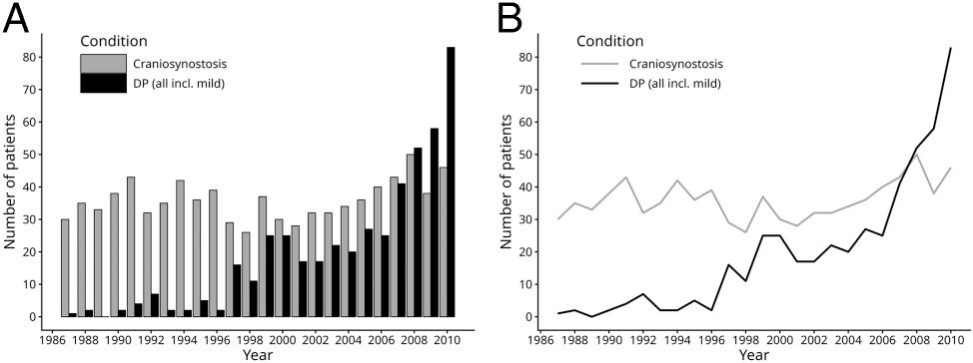
(A, B) Absolute annual numbers of live-born patients with DP and DB (including mild cases; n=466) and craniosynostosis (n=864) in Finland, 1987 to 2010. DP, deformational plagiocephaly; DB, deformational brachycephaly.

Even when mild cases were included, DP and DB diagnoses were rare in Finland between 1987 and 1999, with 79 in 809,681 live births, corresponding to a prevalence of 9.8 per 100,000 live births. In contrast, between 2000 and 2010, 387 cases were recorded among 638,277 live births, yielding a prevalence of 60.6 per 100,000 live births, representing a 6.2-fold increase (+519%), with a significant difference (*P*<0.001).

### Trends in Prevalence of DP and DB Compared With Craniosynostosis

Linear regression analyses and locally estimated scatterplot smoothing (Loess) curves demonstrated a significant increase in DP and DB prevalence from 1987 to 2010, particularly after 2000, and a modest increase in craniosynostosis prevalence during the same period (Fig. 5).

**FIGURE 5:**
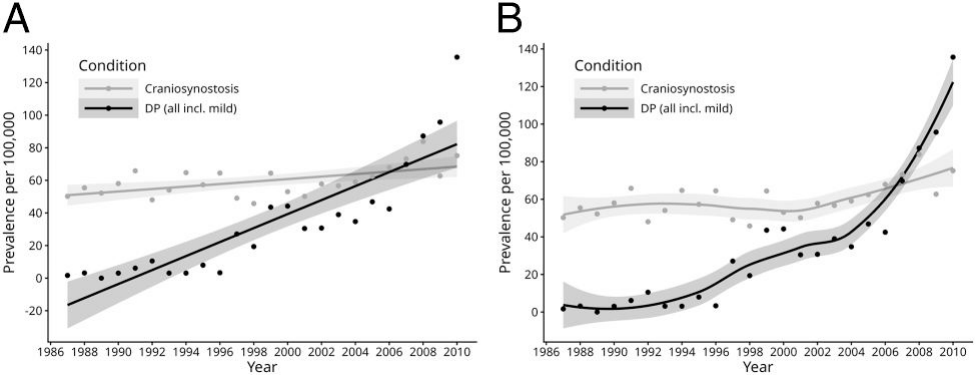
Prevalence per 10,000 live births of DP and DB, including mild cases, and craniosynostosis. The figure presents annual prevalence of DP and DB (black) and craniosynostosis (grey), plotted by year of birth. Dots represent annual data points. Shaded areas indicate 95% confidence intervals. (A) Lines represent linear regression trends. (B) Loess estimates illustrate nonlinear trends. The plots demonstrate a significant increase in DP and DB prevalence, particularly after 2000, and a modest increase in craniosynostosis prevalence. DP, deformational plagiocephaly; DB, deformational brachycephaly; Loess, locally estimated scatterplot smoothing.

## DISCUSSION

### Summary of Findings

This nationwide population-based study highlights a significant increase in the annual prevalence of DP and DB without synostosis in Finland between 1987 and 2010. Between 1987 and 1999, the prevalence began to rise, possibly owing to the introduction of international recommendations for supine infant sleep, although no national campaign was formally launched. The largest shift occurred after 2000, when sleep recommendations in Finland were updated to favor the supine sleep position. Notably, DP and DB prevalence surpassed that of craniosynostosis in the later years of the study, beginning in 2008. This increase was most pronounced in children without additional anomalies. During the same period, the live birth prevalence of craniosynostosis increased modestly across all craniosynostosis groups, including single-suture synostoses, multiple-suture nonsyndromic synostoses (no identified craniosynostosis syndrome), and syndromic craniosynostoses, but was statistically significant only among syndromic patients, as shown in our previous study.^26^

### Comparison With International Trends

Our study confirmed a higher prevalence in males and an increased proportion of twins in the combined group of children with moderate-to-severe deformities.^27,28^ Furthermore, at least 56% of posterior DP cases in this group displayed right-sided occipital flattening, consistent with previous findings attributing this pattern to infant head-positioning preferences.^29^

Similar increases in DP prevalence have been reported following the implementation of infant sleep-positioning guidelines. Kane et al^21^ reported a six-fold increase in referrals within 2 years of the American Academy of Pediatrics’ 1992 recommendation. Similarly, in Finland, we observed a six-fold increase following the introduction of sleep recommendations over a longer period. Branch et al^22^ noted an increase from 9 referrals in 1996 to >600 by 2005. In Finland, a comparable increase was observed, although the absolute numbers remained low owing to the smaller population size. The trend peaked between 2007 and 2010, mirroring international patterns with a delayed onset, and supporting the role of supine sleep positioning as a possible contributing factor to the increasing diagnosis of positional cranial deformities. The patient cohorts reported by Branch and Kane were comparable to our cohort, as they consisted of patients referred to craniofacial centers for evaluation and included children with associated comorbidities.

In a prospective cohort of healthy term infants aged 7 to 12 weeks recruited from routine primary care well-child visits in Alberta, Canada, in 2010 (n=404), Mawji et al^10^ reported that 46.6% of infants had posterior DP and that 78.3% of plagiocephaly cases were classified as mild (Argenta type I), whereas 21.7% were classified as Argenta types II to V. The recommendation for the supine infant sleep position was introduced in Canada in 1999, only slightly earlier than in Finland, and was followed by an increase in plagiocephaly incidence. If this severity distribution was applied to Finland in 2010 (Fig. 2), it is likely that a substantial proportion of mild positional cranial deformities would not have been captured in our registry-based data. However, comparisons are limited by differences in study design and case ascertainment methods. The nationwide recording of primary care diagnoses in Finland began in 2011, limiting the availability of primary care data during the study period.

### Correlation Between Sleep Position Recommendations and SIDS Prevention in Finland

SIDS refers to the unexpected sudden death of an infant aged <1 year that cannot be explained after a thorough case investigation, including a death scene examination, autopsy, and clinical history review.^30^ Its incidence is highest between 1 and 4 months of age, and 90% of SIDS deaths occur within the first 6 months of life.^31^ Supine sleep positioning reduces SIDS rates, especially compared to prone sleeping positioning.^32–34^

Unlike other Nordic countries, Finland has not implemented a broad public health campaign because of its relatively low historical SIDS incidence rate. Between 1969 and 1980, the mean annual SIDS rate in Finland was 0.41 per 1,000 live births.^23^ Although no official nationwide campaign was conducted in Finland, the recommendation for supine infant sleep was gradually adopted in primary care beginning in the 1990s. Sleep position guidelines were formally revised in 2000.^24^ However, implementation progressed slowly, and side sleeping continued to be frequently advised in Finnish maternity clinics in the early 2000s, despite the updated recommendations. The adoption of the supine sleep position became more widespread during the later years of the study. The temporal increases in DP and DB prevalence coincided with this shift, which may reflect the success of sleep position recommendations in Finland.

### Diagnostic Awareness

The early increase in DP and DB diagnoses may have been influenced by growing diagnostic awareness among health care professionals as well as evolving clinical practices and improvements in health care register monitoring systems. Initially, some of these cases were misdiagnosed as lambdoid synostosis, which was reflected in certain congenital malformation surveillance systems as an apparent increase in the incidence of lambdoid synostosis.^35,36^ However, broader recognition of positional skull deformities and advances in imaging modalities, including 3-dimensional computed tomography, magnetic resonance imaging (MRI), and blackbone MRI, have improved the diagnostic accuracy.^37,38^ Our findings are consistent with those of previous studies, underscoring the importance of distinguishing these conditions through accurate clinical and radiologic evaluation.^1,39^

We also observed a modest increase in craniosynostosis prevalence during the study period, which may partly reflect greater overall attention to cranial shape in health care settings, prompted by the increase in deformational head-shape abnormalities. In this study, each patient with DP or DB was evaluated with specific considerations to rule out craniosynostosis, although the assessment was retrospective. No significant increase was observed in the prevalence of lambdoid synostosis or unilateral coronal synostosis in Finland.^26^

The frequency of DP and DB diagnoses among patients with syndromes also increased in our study. In earlier years, cranial asymmetry or occipital flattening may not have been consistently documented in diagnostic codes and registries, particularly among patients treated for other conditions that require prolonged supine positioning. Such cranial deformations may have been considered “normal positional molding” related to the treatment itself. Deformational cranial asymmetry is not a new phenomenon; moreover, it has previously been described in association with other anomalies and treatments.^40^ However, as supine sleep recommendations became more widely adopted and DP and DB gained recognition, these conditions may have received greater clinical attention, reflecting improved diagnostic awareness and documentation rather than a true increase in prevalence.

### Strengths and Limitations of our Study

A major strength of this study is the use of comprehensive, nationwide registry data with linkage across multiple health and hospital registers, complemented by an independent medical record review for diagnostic confirmation and cross-referencing with the registries of the Helsinki University Hospital and Helsinki City Hospital. This approach ensured high case ascertainment accuracy and comprehensive population coverage.

A strong temporal correlation was observed between the gradual adoption of supine sleep recommendations and increases in DP and DB prevalence. However, infant sleep position was not recorded in the Register of Congenital Malformations or other national registries and was inconsistently documented in the hospital records. Therefore, a direct causal relationship between supine sleep and the observed increase in DP and DB could not be established.

The prevalence of DP and DB increased significantly in all the groups, including mild cases. Assessment of mild deformational cases based solely on retrospective medical records was challenging, as subtle cranial flattening can be difficult to distinguish from normal variations in skull shape without direct clinical examination. Consequently, mild cases were excluded from the selected prevalence analyses and additional anomalies were not systematically evaluated in this subgroup.

Furthermore, not all children with DP or DB may have been referred from primary care to secondary or tertiary health care services, particularly those with mild deformities (mild unilateral occipital or symmetric cranial flattening without cranial compensation or facial or ear position asymmetry). Primary care data were not available for this study because the nationwide recording of primary care diagnoses in Finland began in 2011. This may have resulted in overrepresentation of more severe phenotypes and underestimation of the true population prevalence.

In addition, the ICD-10 classification was introduced in Finland in the mid-1990s, and the previously used ICD-9 system did not include a specific diagnostic code for plagiocephaly. Therefore, it is possible that some cases were not coded in earlier years due to the absence of an appropriate diagnostic category.

### Clinical and Public Health Implications

The increasing prevalence of DP and DB has important implications for health care systems and resource utilization in Finland. Although the supine sleep position remains essential for SIDS prevention, its association with cranial deformities should be recognized.^30^ Early infancy, particularly between 1 and 4 months of age, is a critical period in the development of DP.^10,14,41^ This peak period coincides with the highest incidence of SIDS, with approximately 90% of cases occurring before 6 months of age.^31^ Studies in untreated adolescents have reported a substantially lower prevalence of DP than in infancy, suggesting that clinically significant deformity is uncommon later in life.^42^

These findings indicate that cranial asymmetry develops rapidly during the first weeks and months of life and begins to improve spontaneously in most cases from around 3 to 4 months of age. This likely reflects the high malleability of the infant skull and the gradual normalization of head shape with increasing mobility and rapid brain growth.^43^

Important gaps remain in our understanding of the etiology and risk factors of cranial deformities, a topic that has been highlighted in editorials in the *Journal of Craniofacial Surgery*.^44^ Deformational plagiocephaly has been associated with developmental risks; however, these findings do not establish causality and may reflect shared underlying risk factors^45^ or vulnerabilities rather than a direct effect of cranial deformation.^14^ Prematurity is a recognized risk factor, and increased calvarial malleability has been proposed as a contributing factor not only in preterm infants but potentially more broadly, although definitive evidence is lacking.^46^ Vitamin D deficiency during pregnancy and early infancy has also been suggested as a potential risk factor, given its established role in skeletal development;^47^ however, current evidence remains observational.^3^ A better understanding of these pathways is critical for refining preventive strategies, optimizing early developmental surveillance, and guiding the management of DP and DB.

In this context, the role of active interventions, including helmet therapy, should be critically evaluated. Current evidence does not demonstrate an association between helmet therapy and improved neurocognitive, motor, or quality-of-life outcomes, suggesting that these interventions primarily address cranial shape rather than broader functional development.^48–50^ Residual asymmetry may persist even after helmet therapy, particularly in more severe cases and when treatment is initiated later.^48^ For these reasons, helmet therapy is not publicly funded in Finland. Given the potential for spontaneous remodeling, most cases can be managed conservatively.

Potential contributing factors, such as torticollis, hypotonia, head position preference, and conditions requiring prolonged supine positioning, should be identified early in both specialized centers and primary care. Early referral to physiotherapy is recommended, as outcomes are generally favorable when treatment is initiated promptly.^51^

These considerations further underscore the importance of accurate differentiation between deformational plagiocephaly and true craniosynostosis to avoid unnecessary surgical interventions while ensuring timely identification of children with craniosynostosis who require surgical treatment.^1,39^ Cranial vault surgery is generally associated with low mortality but remains a major procedure carrying a risk of perioperative complications.^52^ In addition, recurrence of cranial deformity may occur even after successful cranial vault reconstruction.^53^

Despite its clear benefits in reducing SIDS, concerns about DP may inadvertently reduce adherence to recommended supine sleep practices in some families.^54^ In addition to prone sleeping, side sleeping is also a well-established risk factor for SIDS.^55^ Preventive guidance should therefore reinforce adherence to supine sleep while also providing specific guidance on how to reduce or prevent positional head molding.

A Finnish randomized prospective study conducted in a maternity hospital provided parents of newborns with instructions on varying head position during infant care, reducing time spent in car seats and sitters, and increasing supervised prone positioning (“tummy time”). Infants in the intervention group developed fewer and milder cases of DP than those in the control group.^56^ Mawji et al^2^ suggested that prevention messaging should begin during pregnancy and be reinforced during the postpartum period. Supervised prone positioning is associated with improved motor development, and current WHO recommendations advise at least 30 minutes per day for infants under 1 year of age while awake.^57,58^

Larger prospective cohort studies in pediatric primary care settings are needed to determine the prevalence of DP and DB at specific ages in Finland, particularly during the peak risk period associated with sleep position, which is common to both SIDS and DP. Studies incorporating detailed assessments of potential confounders and parenting practices will be important for guiding effective prevention of both SIDS and DP.

The findings of this study provide a foundation for further investigation of the risk factors, associated anomalies, and developmental outcomes of DP and DB, as well as their relationship with SIDS incidence in Finland. Future population-based studies using updated national registry data, including data from primary care, are needed to determine whether these trends have persisted beyond 2010. Such data are essential for planning and evaluating preventive and clinical strategies, as well as for assessing their impact on the health and quality of life of affected children and their families.

## Supplementary Material
