# Supplementary material for: Prevalence and Temporal Trends of Deformational Plagiocephaly and Brachycephaly in Finland: A Population-Based Register Study
Source: J Craniofac Surg. 2026 May 22;37(10):2710–7. doi: 10.1097/SCS.0000000000012892 (PMC13645403; doi:10.1097/SCS.0000000000012892)
Supplement: Supplementary file 1 [file scs-37-02710-s001.docx]

**Table 1A. Supplemental digital content. Diagnostic codes used in the study**

ICD-10 diagnostic codes:

| ICD-10 code | Diagnosis |
| --- | --- |
| Q75.0 | Craniosynostosis |
| Q75.00 | Coronal synostosis |
| Q75.01 | Sagittal synostosis |
| Q75.02 | Trigonocephaly/Metopic synostosis |
| Q75.03 | Synostosis of multiple sutures |
| Q75.08 | Other synostosis |
| Q75.1 | Crouzon syndrome |
| Q75.2 | Hypertelorism |
| Q75.3 | Macrocephaly |
| Q75.4 | Mandibulofacial dysostosis |
| Q75.5 | Oculomandibular dysostosis |
| Q75.80 | Local skull defect |
| Q75.81 | Frontonasal dysplasia |
| Q75.88 | Other specified craniofacial malformations |
| Q75.9 | Unspecified craniofacial malformation |
| Q87.01 | Apert syndrome |
| Q87.02 | Pfeiffer and Carpenter syndromes |
| Q87.03 | Goldenhar syndrome |
| Q67.2 | Dolichocephaly |
| Q67.3 | Plagiocephaly |
| Q67.4 | Other craniofacial malformations |

ICD-9 diagnostic codes:

| ICD-9 code | Diagnosis |
| --- | --- |
| 7540A | Malformations of the skull, face, maxilla, and mandible |
| 7560A | Craniosynostosis |
| 7560X | Other malformations of the skull and facial bones |
| 7555X | Acrocephalosyndactyly |

† ICD-9 codes were used in Finnish health registers until the mid-1990s and were gradually replaced by ICD-10 codes, which have been in nationwide use since 1996.

**Table 1B. Supplemental digital content. Procedure codes used in the study**

NOMESCO/NCSP (NOMESCO Classification of Surgical Procedures) procedure codes (from 1996):

| NOMESCO code | Procedure |
| --- | --- |
| AAA20 | Placement of a ventricular pressure sensor |
| AAA25 | Placement of an epidural pressure sensor |
| AAA27 | Placement of an intracerebral pressure sensor |
| AAF00 | Ventriculostomy |
| AAF05 | Ventriculoperitoneal shunt |
| AAK00 | Cranioplasty |
| AAK10 | Duraplasty |
| AAK20 | Surgery for craniosynostosis |
| AAK30 | Reconstruction of cranial and facial malformations |
| AAK99 | Other skull and dura surgery |

Finnish Hospital Association (Suomen Sairaalaliitto) procedure codes (from 1983):

| Code | Procedure |
| --- | --- |
| 2123 | Epidural intracranial pressure measurement |
| 2124 | Intracranial pressure measurement |
| 2132 | Ventriculoperitoneal shunt |
| 2192 | Cranioplasty |
| 2193 | Surgery for craniosynostosis |
| 2199 | Other skull surgery |
| 2221 | Revision surgery after cranial procedures |
| 2222 | Hematoma evacuation |
| 2223 | Exploration due to complications |
| 2224 | Other complication-related surgery |

‡ Finnish Hospital Association (Suomen Sairaalaliitto) procedure codes were used until 1997, after which NOMESCO procedure codes were implemented nationwide.
