# Supplementary material for: Prevalence and Temporal Trends of Deformational Plagiocephaly and Brachycephaly in Finland: A Population-Based Register Study
Source: J Craniofac Surg. 2026 May 22;37(10):2710–7. doi: 10.1097/SCS.0000000000012892 (PMC13645403; doi:10.1097/SCS.0000000000012892)
Supplement: Supplementary file 2 [file scs-37-02710-s002.docx]

**TABLE 2. Supplemental digital content. Patient characteristics of the combined group:** moderate to severe posterior DP types II–V, moderate to severe DB types II–III, and anterior DP. Syndromic was defined as having a named syndrome other than craniosynostosis or four or more additional anomalies.

DP deformational plagiocephaly; DB, deformational brachycephaly

|  | All  n (%) | No anomalies n (%) | 1-3 anomalies n (%) | Syndromic  n (%) |
| --- | --- | --- | --- | --- |
|  |  |  |  |  |
| All | **405 (100)** | **250 (61.7)** | **120 (29.6)** | **35 (8.7)** |
| Sex |  |  |  |  |
| Male | 276 (68.1) | 172 (68.8) | 84 (70.0) | 20 (57.1) |
| Female | 129 (31.9) | 78 (31.2) | 36 (30.0) | 15 (42.9) |
|  |  |  |  |  |
| Twin | 27 (6.7) | 20 (8.0) | 5 (4.2) | 2 (5.7) |
|  |  |  |  |  |
| Posterior DP | **364 (89.9)** | **220 (88.0)** | **111 (92.5)** | **33 (94.3)** |
| Right-sided (% of post DP) | 203 (55.8) | 122 (55.5) | 66 (59.5) | 15 (45.5) |
| Left-sided (% of post DP) | 137 (37.6) | 85 (38.6) | 40 (36) | 12 (36.3) |
| Side not recorded (% of post DP) | 24 (6.6) | 13 (5.9) | 5 (4.5) | 6 (18.2) |
|  |  |  |  |  |
| Anterior DP | **13 (3.2)** | **12 (4.8)** | **1 (0.8)** | **0 (0.0)** |
| Right-sided (% of ant DP) | 3 (23.1) | 3 (25.0) | 0 (0.0) | 0 (0.0) |
| Left-sided (% of ant DP) | 9 (69.2) | 8 (66.7) | 1 (100.0) | 0 (0.0) |
| Side not recorded (% of ant DP) | 1 (7.7) | 1 (8.3) | 0 (0.0) | 0 (0.0) |
|  |  |  |  |  |
| DB | **28 (6.9)** | **18 (7.2)** | **8 (6.7)** | **2 (5.7)** |
|  |  |  |  |  |
